# 4D Printing in Biomedical Engineering: Advancements, Challenges, and Future Directions

**DOI:** 10.3390/jfb14070347

**Published:** 2023-06-29

**Authors:** Maziar Ramezani, Zaidi Mohd Ripin

**Affiliations:** 1 Department of Mechanical Engineering, Auckland University of Technology, Auckland 1142, New Zealand; 2 School of Mechanical Engineering, Universiti Sains Malaysia, Nibong Tebal 14300, Malaysia; mezaidi@usm.my

**Keywords:** 4D printing, biocompatibility, biomedical engineering, fabrication techniques, smart materials

## Abstract

4D printing has emerged as a transformative technology in the field of biomedical engineering, offering the potential for dynamic, stimuli-responsive structures with applications in tissue engineering, drug delivery, medical devices, and diagnostics. This review paper provides a comprehensive analysis of the advancements, challenges, and future directions of 4D printing in biomedical engineering. We discuss the development of smart materials, including stimuli-responsive polymers, shape-memory materials, and bio-inks, as well as the various fabrication techniques employed, such as direct-write assembly, stereolithography, and multi-material jetting. Despite the promising advances, several challenges persist, including material limitations related to biocompatibility, mechanical properties, and degradation rates; fabrication complexities arising from the integration of multiple materials, resolution and accuracy, and scalability; and regulatory and ethical considerations surrounding safety and efficacy. As we explore the future directions for 4D printing, we emphasise the need for material innovations, fabrication advancements, and emerging applications such as personalised medicine, nanomedicine, and bioelectronic devices. Interdisciplinary research and collaboration between material science, biology, engineering, regulatory agencies, and industry are essential for overcoming challenges and realising the full potential of 4D printing in the biomedical engineering landscape.

## 1. Introduction

The advent of 3D printing technology has transformed various fields, including biomedical engineering, by allowing the fabrication of complex structures with high precision and accuracy [1]. Over the years, 3D printing has been employed to create patient-specific implants, prosthetics, and even living tissue constructs for regenerative medicine [2,3,4,5]. However, these static structures lack the dynamic functionality needed to mimic the behaviour of living systems. The emergence of 4D printing, a technology that combines 3D printing with smart materials that can change shape or properties over time in response to external stimuli, has opened new possibilities for creating dynamic structures in biomedical engineering. 4D printing technology incorporates stimuli-responsive materials, such as shape-memory polymers, hydrogels, and bio-inks, into the printing process to create structures capable of transforming under specific conditions, such as changes in temperature, pH, or moisture [6,7]. These dynamic materials enable the design of advanced biomedical devices that can adapt to the physiological environment, leading to improved therapeutic outcomes and patient-specific treatments.

### 1.1. Brief Overview of 3D Printing in Biomedical Engineering

3D printing, also known as additive manufacturing, has become an indispensable tool in the field of biomedical engineering over the past few decades. This technology allows for the creation of three-dimensional objects by depositing materials layer-by-layer based on a digital model [8,9]. The versatility of 3D printing has led to its widespread adoption in various biomedical applications, from patient-specific implants and prosthetics to tissue engineering and drug delivery systems.

One significant application of 3D printing in biomedical engineering is the fabrication of patient-specific implants and prosthetics [10]. Custom-made implants, such as cranial and dental implants [11], have demonstrated improved clinical outcomes and patient satisfaction by ensuring a precise fit and reducing surgical complications. Similarly, 3D-printed prosthetics have enabled the rapid and cost-effective production of personalised devices, improving patient comfort and functionality.

Tissue engineering and regenerative medicine have also benefited from 3D printing technology. By utilising biocompatible materials, such as hydrogels and bio-inks [12,13], researchers have been able to create complex tissue constructs with specific architectures that mimic native tissues. These constructs can serve as scaffolds for cell growth and differentiation, with the ultimate goal of creating functional tissues and organs for transplantation and disease modelling.

Furthermore, 3D printing has enabled the development of innovative drug delivery systems by allowing for precise control over the shape, size, and composition of drug carriers, such as microcapsules, microneedles, and tablets. These customisable systems can improve drug release kinetics, enhance bioavailability, and enable personalised dosing [14].

Despite the significant advancements achieved with 3D printing in biomedical engineering, there is a growing need for dynamic, stimuli-responsive structures that can better replicate the complex behaviour of living systems. The emergence of 4D printing technology has the potential to address this need and further expand the capabilities of additive manufacturing in the biomedical field.

### 1.2. Definition of 4D Printing

4D printing is an advanced form of additive manufacturing that combines the principles of 3D printing with the use of smart materials that are capable of changing their shape, properties, or functionality over time in response to external stimuli [15]. The term “4D” refers to the fourth dimension, which is time, emphasising the dynamic behaviour of these printed structures. By incorporating stimuli-responsive materials, such as shape-memory polymers, hydrogels, and bio-inks, 4D printing technology enables the creation of dynamic structures that can adapt to their environment, offering new possibilities for biomedical applications [16].

The significance of 4D printing in biomedical engineering lies in its potential to transform various aspects of healthcare, including tissue engineering, drug delivery systems, medical devices, and diagnostics. The dynamic nature of 4D-printed structures allows for the development of more sophisticated and adaptive devices that can mimic the complex behaviour of living systems, ultimately improving therapeutic outcomes and enabling patient-specific treatments [17].

For instance, in tissue engineering and regenerative medicine, 4D printing can be used to create constructs that transform into the desired shape or structure upon implantation, facilitating better integration with the surrounding tissue and promoting cell growth and differentiation [17]. Similarly, 4D printing can enable the development of adaptive drug delivery systems that release their payload in response to specific physiological triggers, such as changes in pH or temperature, providing more targeted and controlled drug delivery [15].

Moreover, 4D printing has the potential to enhance the performance of medical devices and prosthetics by enabling self-adjusting, self-assembling, or self-healing capabilities, improving patient comfort, and ensuring a more precise fit. In diagnostics, 4D-printed sensors and devices could adapt to various biological conditions, providing more accurate and reliable detection of biomarkers, pathogens, or other analytes [18,19].

**Figure 1:**
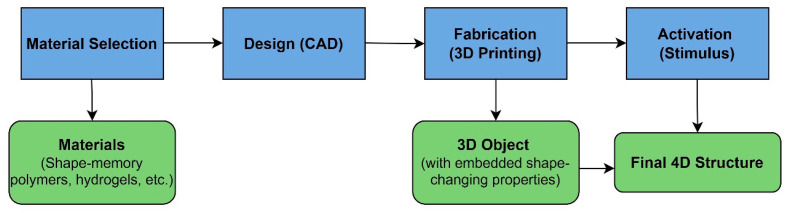
An overview of the 4D printing process.

Figure 1 shows an overview of the 4D printing process, which includes the stages of material selection, design, fabrication, and activation. The material selection stage involves choosing the appropriate stimuli-responsive materials that can undergo controlled shape transformations. These materials could be shape-memory polymers, hydrogels, or other stimuli-responsive composites. In the design stage, the object’s 3D model is designed using computer-aided design (CAD) software. The design incorporates the material’s properties and the desired shape transformations, taking into account factors such as stress, strain, and the specific stimulus. The designed 3D model is fabricated using a 3D printer, which deposits or cures the selected material layer-by-layer, creating a 3D object with embedded shape-changing properties. The printed object is exposed to a specific stimulus, such as heat, light, or moisture, triggering the shape transformation and resulting in the final 4D structure.

Table 1 compares the key features, advantages, and limitations of 3D and 4D printing technologies in the context of biomedical engineering. Overall, the advent of 4D printing technology holds significant promise for advancing the field of biomedical engineering by enabling the development of dynamic, stimuli-responsive structures that better replicate the complexity of living systems and address unmet clinical needs.

### 1.3. Objectives of the Paper

The primary objective of this review paper is to provide a comprehensive and up-to-date overview of the advancements, challenges, and future directions in the field of 4D printing for biomedical engineering applications. As 4D printing technology continues to evolve, it is crucial for researchers, engineers, and clinicians to understand its implications, opportunities, and limitations in the biomedical field. To achieve this objective, the review paper is structured to provide a detailed analysis of the advancements in 4D printing for biomedical applications, including the development of smart materials, such as stimuli-responsive polymers, shape-memory materials, and bio-inks, and the various fabrication techniques employed, such as direct-write assembly, stereolithography, and multi-material jetting.

A thorough exploration of the various biomedical applications of 4D printing, including tissue engineering and regenerative medicine, drug delivery systems, medical devices and prosthetics, and diagnostic tools will be conducted, as well as an examination of the challenges associated with 4D printing in biomedical engineering, such as material limitations related to biocompatibility, mechanical properties, and degradation rates; fabrication complexities arising from the integration of multiple materials, resolution and accuracy, and scalability; and regulatory and ethical considerations surrounding safety, efficacy, and intellectual property. A discussion of the future directions and potential impact of 4D printing on the biomedical engineering field will also be presented, emphasising the need for material innovations, fabrication advancements, and emerging applications such as personalised medicine, nanomedicine, and bioelectronic devices.

By providing a comprehensive overview of the current state and prospects of 4D printing in biomedical engineering, this review paper aims to serve as a valuable resource for researchers, engineers, and clinicians interested in harnessing the potential of this transformative technology to improve healthcare and patient outcomes.

## 2. Advancements in 4D Printing for Biomedical Applications

Over the last few years, advancements in 4D printing have significantly impacted the field of biomedical applications. This innovative technology has the potential to reshape the biomedical sector, offering promising solutions for various challenges. In the following sections, we will discuss various materials and manufacturing processes employed for 4D printing. We will then discuss an array of biomedical applications for 4D printing.

### 2.1. Materials and Manufacturing Techniques for 4D Printing

Materials and manufacturing techniques for 4D printing are essential for achieving the desired shape-shifting and functionality in printed objects. In 4D printing, smart materials are utilised due to their ability to change properties under specific environmental conditions or stimuli. Advanced manufacturing techniques for 4D printing enable the precise fabrication of complex, multi-material structures with tailored responses to specific stimuli. These techniques have evolved to meet the unique requirements of 4D printing, including the ability to integrate smart materials and achieve high-resolution features. A brief overview of these materials and techniques can be found in the subsequent sections.

#### 2.1.1. Smart Materials for 4D Printing

The development of smart materials that can respond to external stimuli is a critical aspect of 4D printing in biomedical engineering. These materials enable the creation of dynamic structures capable of transforming their shape or properties in response to changes in the environment. Incorporating smart materials into 4D printing processes allows researchers to create dynamic structures with transformative potential across the healthcare field.

Shape-memory polymers (SMPs) are one example of smart materials used in 4D printing. SMPs can transition between a deformed state and their original shape upon exposure to a specific stimulus, such as heat or light [20]. This property enables the fabrication of 4D-printed structures that change their shape or configuration when subjected to the appropriate trigger [21,22,23]. For instance, an SMP-based 4D-printed scaffold can adapt its shape to better fit the tracheal structure when exposed to body heat, potentially improving the effectiveness and precision of tracheal repair procedures [24].

Hydrogels are another example of smart materials in 4D printing. These highly absorbent polymer networks can swell or shrink in response to changes in their surrounding environment, such as temperature or pH [6,25]. Hydrogels have been employed in various biomedical applications, including drug delivery systems and tissue engineering. For example, a 4D-printed hydrogel-based drug delivery system can release medication at a controlled rate when exposed to specific temperature levels, ensuring targeted and precise drug administration [26].

Thermo-responsive polymers are a class of smart materials used in 4D printing that exhibit significant changes in their physical properties, such as shape, stiffness, or volume, upon exposure to varying temperatures [27,28,29]. For example, Wang et al. [27] explored the potential of dual thermo-responsive polymer systems to minimise thermal damage to surrounding healthy tissues during photothermal therapy, which is commonly used to treat various diseases, including cancer. The system consisted of two distinct thermo-responsive polymers with different phase transition temperatures. By carefully selecting and combining these polymers, they created a complex structure with tunable thermal properties. By employing 4D printing techniques, complex structures composed of thermo-responsive polymers with tunable properties can be fabricated. These structures can undergo a thermo-induced phase transition in response to external stimuli, such as laser irradiation, enabling precise control over heat generation and distribution. The use of these 4D-printed thermo-responsive polymer systems offers a novel approach to enhance the accuracy and safety of photothermal therapy, reducing the risk of collateral damage to healthy tissues and improving therapeutic outcomes in biomedical applications.

Electroactive polymers and magnetically responsive materials, which can alter their shape or properties in response to electrical stimulation or magnetic fields, respectively, are examples of smart materials used in 4D printing with significant potential in the biomedical field. These materials have been employed in the development of advanced medical devices, such as soft robotic prosthetics and targeted drug delivery systems. By harnessing their unique properties, researchers are exploring novel applications in areas such as tissue engineering, bio-inspired devices, and personalised healthcare, paving the way for innovative solutions to complex biomedical challenges [25,30,31]. For instance, Zhao et al. [31] developed a personalised tracheal scaffold that can mimic the dynamic behaviour of the natural trachea. To achieve this, they utilised 4D printing techniques and shape-memory composites that respond to magnetic stimulation. By incorporating magnetic particles into the shape-memory composites, they created a tracheal scaffold that can undergo controlled shape changes when subjected to external magnetic fields. This magnetic responsiveness allows the scaffold to dynamically adapt and respond to physiological conditions or external stimuli, replicating the natural behaviour of the trachea. The personalised nature of 4D printing enables the fabrication of patient-specific tracheal scaffolds, tailoring the design and dimensions to the individual’s anatomy.

In addition to these smart materials, bio-inks play a crucial role in 4D printing applications, particularly in the realm of biomedical engineering. Bio-inks are biocompatible materials that often contain living cells, allowing for the creation of structures that can transform and develop into functional tissue over time. By combining bio-inks with the dynamic capabilities of 4D printing, researchers can fabricate complex, multi-layered structures that mimic the natural architecture of human tissues [32,33,34]. For instance, a skeletal muscle model has been developed using a cell-aligned bio-ink processed with an electric-field assisted 3D/4D bioprinting technique, resulting in a functional muscle tissue construct, offering potential advancements in the field of tissue engineering and regenerative medicine for muscle repair or replacement [35].

For polymer-based additive manufacturing, the rheological properties of polymers play a significant role in the success and feasibility of 4D printing for biomedical applications [36]. The flow behaviour of polymers during the printing process determines their ability to be extruded, deposited, and retain shape fidelity. The viscosity, shear thinning behaviour, and thixotropic properties of the polymer matrix affect its flowability and the resolution of printed structures. Controlling these rheological properties is crucial for achieving precise and accurate printing outcomes, especially when dealing with intricate geometries or delicate structures. Moreover, the choice of polymer rheology can influence the mechanical performance, biocompatibility, and degradation characteristics of the printed constructs, which are vital considerations for biomedical applications. Therefore, a deep understanding of polymer rheology and its impact on the 4D printing process is essential for optimising the fabrication of functional and biologically relevant structures in the field of biomedical engineering.

In 4D printing for biomedical applications, a wide range of materials are being explored and utilised to achieve desired functionalities and performance. These materials can be categorised into different types, including herbal, bio-based, synthetic, and hybrid materials. Herbal materials, derived from natural sources such as plants or traditional medicines, offer the potential for biocompatibility, biodegradability, and the incorporation of bioactive compounds. Bio-based materials, typically derived from renewable resources, encompass polymers, hydrogels, and composites that exhibit biocompatibility and can mimic the extracellular matrix of tissues. Synthetic materials, such as thermoplastics and shape-memory polymers, offer high mechanical strength, durability, and precise control over shape-memory properties. These materials can be tailored to meet specific requirements, including biocompatibility, degradation rates, and mechanical properties. Hybrid materials combine different types of materials, such as combining natural polymers with synthetic additives or incorporating bioactive agents into synthetic matrices. These hybrids can enhance properties such as mechanical strength, bioactivity, and controlled release capabilities. The choice of material in 4D printing for biomedical applications depends on factors such as the desired functionality, biocompatibility, degradation profile, mechanical requirements, and the targeted tissue or organ. Researchers continue to explore and develop new materials, as well as optimise existing ones, to expand the possibilities and applications of 4D printing in the field of biomedical engineering.

#### 2.1.2. Fabrication Techniques for 4D Printing

Various fabrication techniques have been adapted for 4D printing, with each method offering specific advantages and limitations depending on the materials used and the desired application. Below are the typical manufacturing processes used for 4D printing.

**Figure 2:**
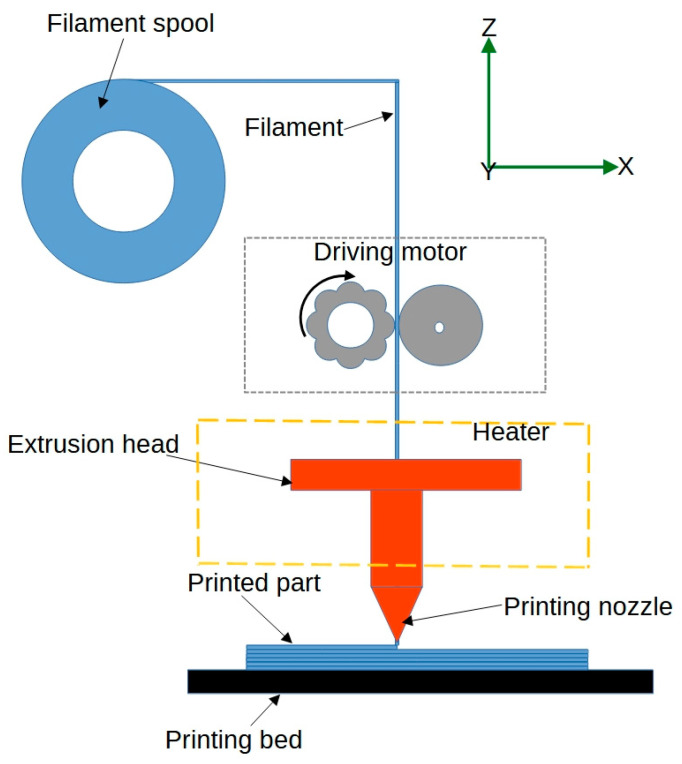
Schematics of the fused deposition modelling process.

*Fused Deposition Modelling (FDM)* is a widely used 3D printing technique that involves the extrusion of a thermoplastic filament through a heated nozzle, which deposits the material layer by layer to create the desired object [37]. Figure 2 shows a schematic representation of the FDM process. In 4D printing for biomedical engineering, FDM can be used to print structures with shape-memory polymers, which can change their shape upon exposure to specific stimuli, such as heat [38]. For example, researchers have used FDM to print customisable lattice structures for bone implants. This innovative approach enables the implant to conform to a patient’s unique anatomy, which may enhance osseointegration, improve surgical outcomes, and increase patient comfort [39].

**Figure 3:**
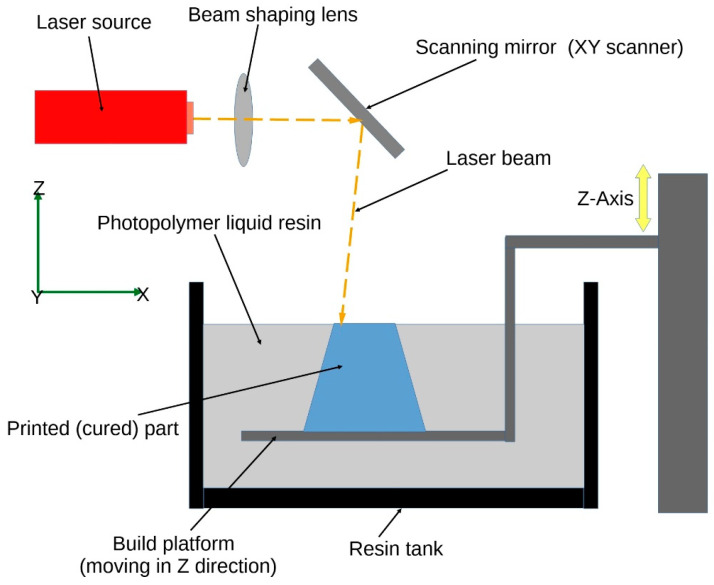
Schematic representation of the stereolithography process.

*Stereolithography (SLA)* is a 3D printing technique that uses a light source, usually a laser, to selectively cure a photosensitive resin layer by layer [40]. A sketch of the stereolithography process is depicted in Figure 3. By incorporating smart materials into the resin, researchers can create 4D-printed structures with biomedical applications. For instance, scientists have used SLA to print hydrogels that swell or shrink in response to changes in pH, which could be used in drug delivery systems for controlled and targeted release of medications [41,42].

**Figure 4:**
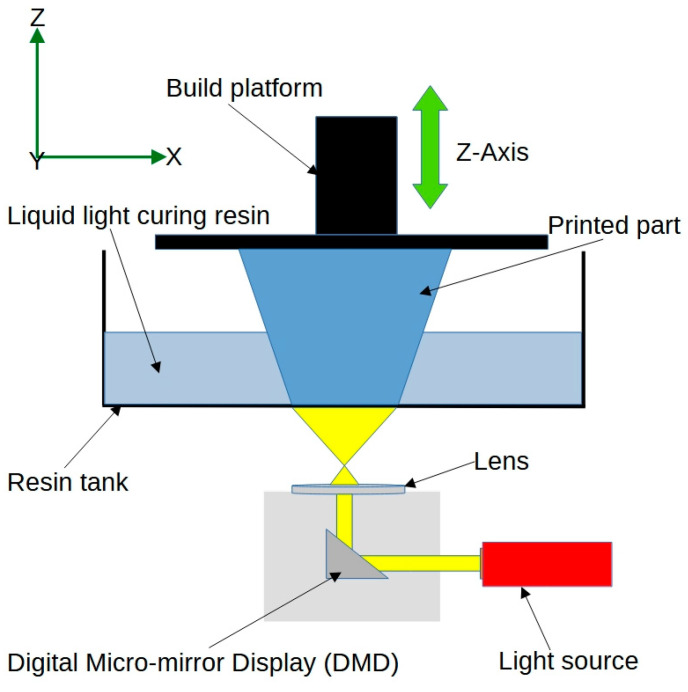
Schematic view of the digital light processing technique.

*Digital Light Processing (DLP)* is similar to SLA, but instead of using a laser, it employs a digital light projector to cure the resin. Figure 4 shows a schematic view of the DLP process. This technique can achieve higher printing speeds and finer resolution compared to SLA, making it suitable for printing intricate 4D structures in biomedical engineering [43]. Researchers have used DLP to create 4D-printed bioresorbable devices with near-infrared responsiveness, photothermal properties, and shape-memory functions. These innovative devices have the potential to transform various medical applications, such as drug delivery and minimally invasive surgery, by offering customisable, dynamic, and efficient solutions [44].

**Figure 5:**
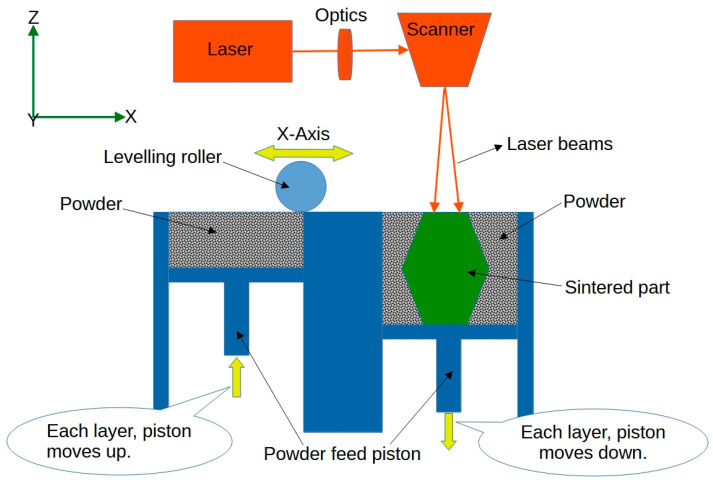
Sketch of the selective laser sintering process.

*Selective Laser Sintering (SLS)* is a powder-based 3D printing technique that uses a laser to selectively sinter or fuse powdered material layer by layer, as illustrated in Figure 5. By incorporating shape-memory polymers or other smart materials in the powder, it is possible to create 4D-printed structures for biomedical applications [45]. For example, Mei et al. [46] used SLS 4D printing to develop shape-memory thermoplastic polyamide elastomers that demonstrated the ability to change their shape in response to heat. These materials possess shape-memory properties, allowing them to undergo significant deformations and retain a temporary shape. By precisely controlling the printing parameters and the heating–cooling cycles, the researchers demonstrate the ability to create complex 3D-printed objects that exhibit shape-memory behaviour. It has promising applications in biomedical areas. One example is in the field of tissue engineering, where 4D-printed scaffolds can be designed to change shape and adapt to specific anatomical sites within the body. These scaffolds can be implanted in a temporary shape, and upon exposure to body temperature or other stimuli, they can transform into their intended shape, promoting tissue regeneration and integration.

**Figure 6:**
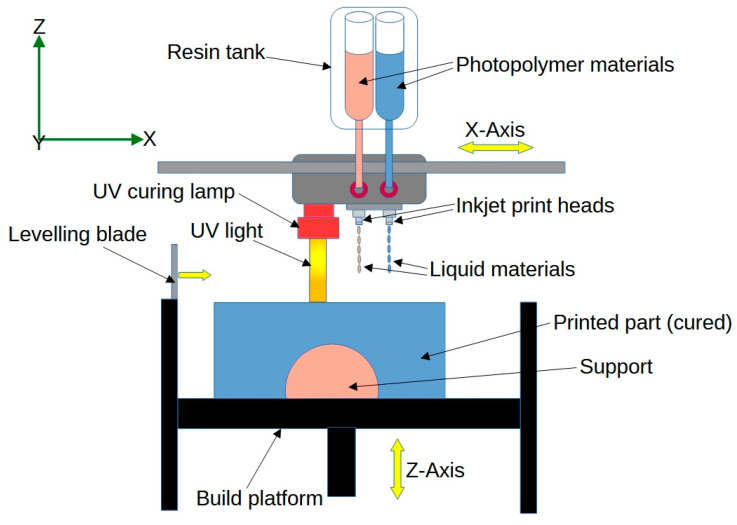
Schematic of the multi-material jetting process.

*Multi-Material Jetting* involves the simultaneous deposition of multiple materials during the printing process, allowing for the creation of 4D-printed structures with varying mechanical properties or responsiveness to stimuli [47]. A schematic presentation of the multi-material jetting technique is shown in Figure 6. Multi-material jetting enables the fabrication of complex, multi-functional objects for biomedical applications. Researchers have used this technique to manufacture liquid–solid co-printing of multi-material 3D fluidic devices with potential biomedical applications such as microfluidic devices or lab-on-a-chip systems [48].

**Figure 7:**
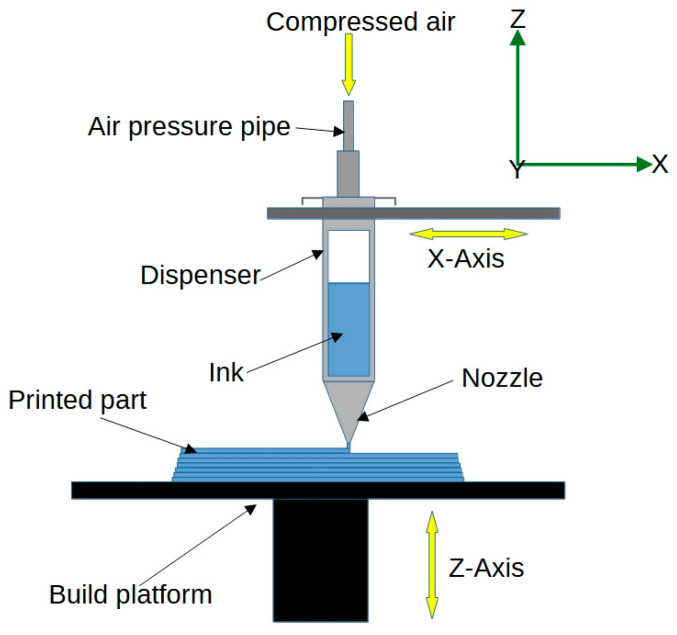
Schematic representation of the direct ink writing process.

*Direct Ink Writing (DIW)* is an extrusion-based printing technique that involves the deposition of a viscous ink, often containing particles or fibres, to create the desired structure. A schematic view of the DIW process is shown in Figure 7. In 4D printing for biomedical applications, DIW can be used to print structures using bio-inks containing living cells, enabling the fabrication of functional tissue constructs for regenerative medicine and tissue engineering [49,50]. For example, scientists have used DIW-based 4D printing to fabricate smart hydrogel scaffolds that can undergo controlled shape transformations in response to specific environmental stimuli [51]. This approach has the potential to enhance the fabrication of patient-specific implants, improving patient outcomes, and advancing the fields of drug delivery and minimally invasive surgery.

The successful implementation of additive manufacturing techniques relies on carefully selecting the operational conditions and process parameters, taking into account a multitude of factors such as material selection, geometry of the printed object, and the intended final application. Each additive manufacturing technique requires tailored settings to achieve optimal results. Variables such as print speed, layer thickness, temperature, and energy input must be optimised to ensure the proper adhesion, dimensional accuracy, and mechanical properties of the printed object. Furthermore, factors such as support structures, post-processing requirements, and even environmental conditions may influence the choice of operational conditions. Considering the unique combination of material properties, geometry, and desired application is crucial to determine the most suitable parameters, resulting in high-quality, functional, and customised additive manufacturing outcomes.

### 2.2. Applications of 4D Printing in Biomedical Engineering

**Figure 8:**
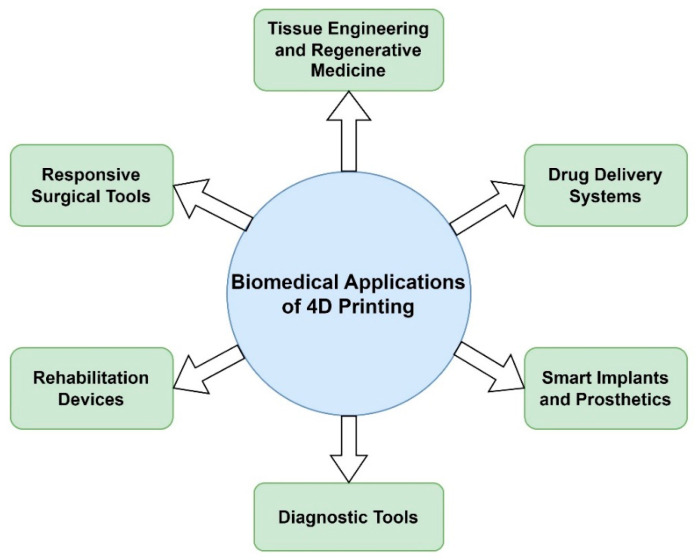
Selected biomedical applications of 4D printing.

Figure 8 serves as a visual summary of the various biomedical applications of 4D printing technology that will be thoroughly discussed in the subsequent sections, offering a comprehensive overview of the potential impact of this technology on the future of healthcare.

#### 2.2.1. Smart Implants and Prosthetics

The emergence of 4D printing technology has brought about remarkable progress in the creation of smart implants and prosthetics. By adding the element of time as the fourth dimension, 4D-printed devices can self-transform or adapt in response to specific stimuli, such as temperature, light, moisture, or magnetic fields. This unique attribute has led to a significant shift in the field of biomedical engineering, particularly in the design and fabrication of customisable and adaptable implants and prosthetics.

One example of customisation and adaptability in smart implants and prosthetics is the development of personalised dental implants [45]. By using 4D printing technology, dental professionals can create customised implants that perfectly fit the patient’s unique oral structure and even adapt to changes in the jawbone over time. This level of customisation and adaptability ensures optimal functionality, reduced discomfort, and improved patient satisfaction. Another noteworthy example of 4D printing technology in orthopaedics surgery is the creation of customised, patient-specific implants. These 4D-printed implants can conform to the unique anatomical structures of each patient, ensuring a precise fit and reducing the risk of complications. The use of smart materials and adaptive designs allows the implants to adjust to the patient’s body changes over time, minimising the need for further adjustments [52,53].

4D-printed smart implants and prosthetics have the potential to reduce the need for invasive surgeries and adjustments. Traditional implants and prosthetics often necessitate multiple surgical interventions for installation, replacement, or adjustments, which can be physically and emotionally taxing for patients. In contrast, 4D-printed devices can adapt and adjust themselves according to the body’s changing needs [54,55,56]. For instance, a 4D-printed intestinal stent can be designed with near-body temperature-triggered shape-memory biocomposites to respond to changes in the patient’s internal environment, allowing them to adjust their shape accordingly to provide optimal support for the affected intestinal region [57]. Additionally, 4D-printed shape-memory vascular stents using βCD-g-Polycaprolactone can automatically adjust their shape in response to changes in blood flow or vessel diameter, providing optimal support for the affected blood vessel and minimising the risk of complications. The stents’ self-adjusting capabilities reduce the need for additional surgical interventions to replace or modify the stent, thus enhancing patient outcomes and streamlining the treatment process [58].

Smart implants can be designed to change their shape or functionality over time, allowing them to accommodate the patient’s needs better. For example, a 4D-printed spinal implant can gradually change its shape to support the spine as it heals, potentially reducing the risk of complications and promoting faster recovery [59,60].

#### 2.2.2. Drug Delivery Systems

4D printing technology has demonstrated remarkable potential in the development of advanced drug delivery systems. These systems offer significant advantages over traditional methods, including controlled release of medication, patient-specific dosing, and targeted delivery, ultimately leading to improved treatment outcomes and patient experiences [61].

Controlled release of medication is a vital aspect of modern drug delivery systems. By utilising 4D-printed materials that respond to specific stimuli, such as temperature or pH changes, researchers can create devices capable of releasing drugs at a precise rate over a predetermined period [62]. For example, a 4D-printed hydrogel capsule could be designed to release its contents gradually in response to the acidic environment of the stomach. This controlled release helps maintain consistent drug concentrations in the body, reducing the risk of side effects and improving therapeutic efficacy [63].

Another example is the development of 4D-printed, personalised drug-eluting implants for cancer treatment. These implants can be designed to release chemotherapeutic agents at a controlled rate in response to specific triggers, such as changes in pH or the presence of particular biomarkers. This targeted drug delivery approach ensures that patients receive the appropriate dosage of medication precisely when and where it is needed, minimising the risk of side effects and increasing the overall effectiveness of the treatment [64].

Patient-specific dosing and targeted delivery are crucial aspects of advanced drug delivery systems. Utilising the potential of 4D printing technology, researchers can develop devices customised to individual patients’ requirements, ensuring optimal dosing and minimising adverse effects. For example, a bioinspired 4D-printed hydrogel capsule designed for smart controlled drug release can respond to environmental stimuli, such as changes in pH or temperature, and release the medication accordingly [65]. Additionally, a 4D-printed microneedle patch could be used for personalised pain management, releasing analgesics in response to inflammation or pain signals. This innovative approach offers a non-invasive and patient-specific treatment method, minimising side effects and improving overall therapeutic outcomes [66].

By creating 4D-printed drug carriers that react to particular cellular or environmental triggers, researchers can ensure that medication is directly administered to the intended site of action. An example is the utilisation of 4D printing to design smart gastroretentive, esophageal, and intravesical drug delivery systems [62]. These 4D-printed devices can adapt their shape or release profiles in response to specific physiological conditions, such as contact with certain tissue types. This precise targeting minimises the impact on healthy tissues and optimises the effectiveness of the delivered medication.

#### 2.2.3. Tissue Engineering and Regenerative Medicine

4D printing technology has shown immense promise in the fields of tissue engineering and regenerative medicine [67,68]. By incorporating living cells into bio-inks and leveraging the unique capabilities of 4D printing, researchers have been able to create structures that can transform and develop into functional tissue over time. This ground-breaking approach has the potential to transform organ transplantation and promote faster healing for damaged tissues.

Bio-inks, which are biocompatible materials containing living cells, are a critical component of 4D printing in tissue engineering [69]. By combining these bio-inks with the dynamic capabilities of 4D printing, scientists can fabricate complex, multi-layered structures that mimic the natural architecture of human tissues [70].

For example, cartilage tissue can be 4D-printed using a bio-ink containing human mesenchymal stromal cells and a swelling-dependent hydrogel [71]. Upon swelling, the printed construct undergoes shape-based transformation, closely mimicking the natural cartilage structure. Additionally, a 4D cell condensate bioprinting technique has been developed to facilitate the creation of tissue constructs with precise control over cellular organisation and spatial distribution [72]. This approach allows for the production of customised tissue structures that can adapt and respond to specific environmental cues. These innovations provide a potential solution for cartilage repair or replacement, paving the way for advancements in tissue engineering and regenerative medicine.

**Figure 9:**
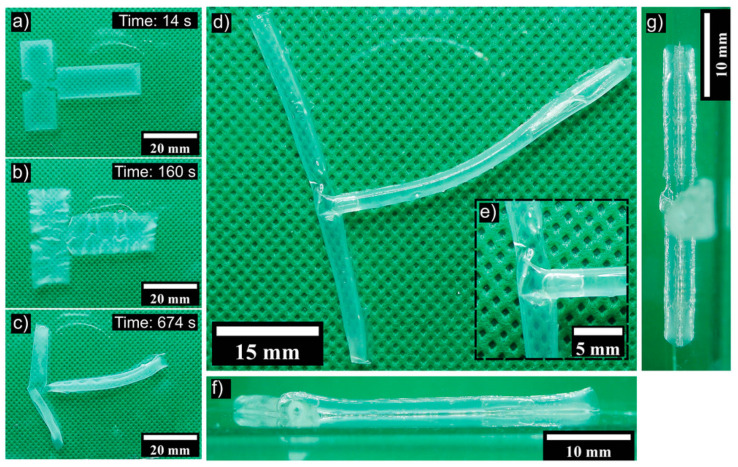
Illustration of the self-folding process of 3D-printed structures and the formation of a T-junction. (**a**) The initial state of the dried and UV crosslinked hydrogel immediately after water addition. (**b**) Swelling and detachment of the sample from the substrate. (**c**) Formation and folding of the T-junction. (**d**) The final T-junction after water removal and drying. (**e**) A zoomed view of the junction area. (**f**,**g**) Side views of the structure [73].

In addition to cartilage, 4D printing has also been used to create functional blood vessels. By utilising a bio-ink containing endothelial cells (which form the lining of blood vessels), researchers have successfully 4D-printed sophisticated structures with potential applications in vascularised tissues. An example is the 4D biofabrication of T-shaped vascular bifurcation, which is a critical component of blood vessel networks [73]. Figure 9 shows that a 3D-printed T-junction structure undergoes controlled shape transformation due to swelling of the polymer. The printed structure is designed to respond to specific environmental cues, resulting in the formation of a complex vascular bifurcation that can be integrated into engineered tissues. This innovative approach holds significant potential for the creation of vascularised tissues, which are crucial for the survival and function of larger engineered organs.

The potential for organ transplantation alternatives is a significant area of interest within the realm of tissue engineering and regenerative medicine. Due to the severe shortage of donor organs and the risks associated with transplantation, such as rejection and infection, there is a pressing need for alternative solutions [74]. 4D printing offers a promising approach by enabling the creation of patient-specific, functional organs that can adapt and integrate with the recipient’s body [75].

For instance, A 3D-printed medical model company collaborated with surgeons at Belfast City Hospital in Northern Ireland to successfully create a functional 3D-printed replica of the kidney [76]. By replicating the complex architecture of the kidney and incorporating living cells, the printed structures could potentially develop into functional organs that can be transplanted to replace damaged or failing kidneys, significantly improving patients’ quality of life.

#### 2.2.4. Responsive Surgical Tools

4D printing technology has shown immense potential in the development of responsive surgical tools that can adapt to changes in their environment, providing surgeons with greater precision and control during complex procedures. By leveraging the unique properties of 4D-printed materials, these innovative tools can adjust their shape and stiffness in response to specific stimuli, leading to improved surgical outcomes and reduced recovery times for patients [77].

**Figure 10:**
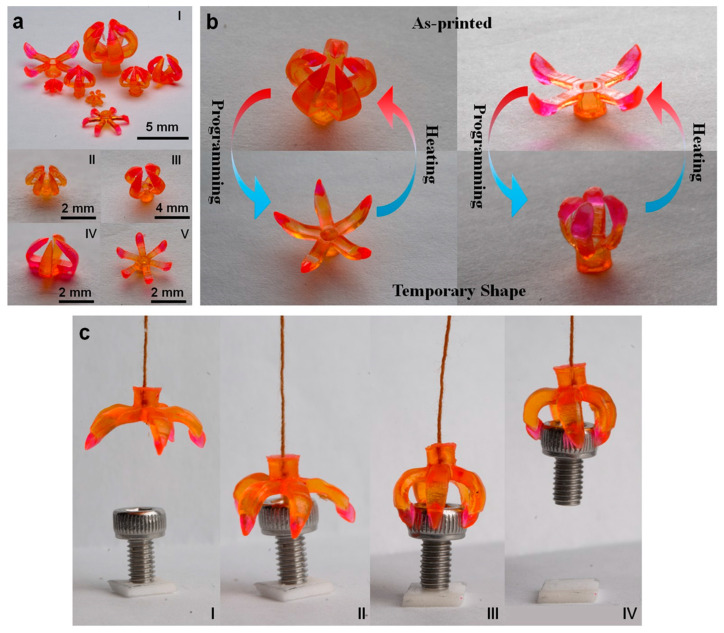
(**a**) Various designs of multimaterial grippers fabricated for the study. (**b**) Illustration depicting the transition from the printed shape to the temporary shape of multimaterial grippers. (**c**) Sequential snapshots capturing the process of gripping an object using the multimaterial grippers [78].

Shape and stiffness adaptability is a crucial aspect of responsive surgical tools, as it allows them to conform to different surgical scenarios and anatomical structures, minimising the risk of damage to surrounding tissues [77]. For example, a 4D-printed surgical gripper has been fabricated that changes its shape and stiffness in response to environmental stimuli within the body, allowing it to navigate through tight spaces, grasp and manipulate delicate tissue, and minimise the risk of damage during surgical procedures [78]. Figure 10 showcases the 3D printing process of a structure incorporating multiple shape-memory polymers. These multimaterial grippers exhibit promising capabilities as microgrippers for object manipulation and as drug-delivery devices capable of controlled object release [78]. Another example of 4D printing of responsive surgical tools is a 4D-printed self-expanding stent that can change its diameter based on the patient’s specific needs. This stent can be used during endovascular procedures to ensure optimal blood flow and adapt to changes in vessel diameter or pressure over time, reducing the risk of complications such as in-stent restenosis [79].

Improved surgical outcomes and reduced recovery times are important benefits of utilising responsive surgical tools. By providing surgeons with greater control and adaptability during procedures, these 4D-printed devices can help minimise the risk of complications, such as bleeding, infection, or damage to adjacent structures. For example, a 4D-printed microneedle array inspired by the backward-facing barbs found in some natural structures, such as porcupine quills, could be developed to enhance tissue adhesion during medical procedures. These microneedles, when inserted into the tissue, would have barbs that open and lock into the tissue, providing a more secure and stable connection. This innovative microneedle array could potentially improve the performance of transdermal drug delivery systems or be used in wound closure applications, minimising the risk of dislodgement and ensuring more effective treatment outcomes [80]. Furthermore, the use of responsive surgical tools can also contribute to shorter recovery times by minimising tissue trauma and promoting faster healing. For example, a 4D-printed wound closure device that can adapt its shape and stiffness to the wound’s contours could provide more precise and gentle closure, resulting in reduced scarring and faster healing [81].

#### 2.2.5. Diagnostic Tools

4D printing technology has shown great potential in the development of advanced diagnostic tools that can revolutionise the field of medical diagnostics. By leveraging the unique properties of 4D-printed materials, which can change their shape or properties in response to specific stimuli, researchers can create diagnostic devices that are more sensitive, accurate, and patient-specific, ultimately leading to improved patient care and outcomes.

One example of a 4D-printed diagnostic tool is a wearable smart sensor created using thermoplastic polyurethane (TPU) printed on fabric. This sensor can monitor vital signs, such as heart rate, blood pressure, and body temperature, by utilising the stimuli-responsive properties of the TPU material. For instance, a 4D-printed temperature sensor can change its shape or electrical properties in response to fluctuations in body temperature, providing an accurate and continuous measurement that can help detect fever or inflammation. This innovative wearable sensor can enable real-time monitoring and early detection of potential health issues, paving the way for personalised healthcare solutions [82].

Another example of a 4D-printed diagnostic tool is a solid-cured tissue-engineered implant made from photo-polymerisable resins. These resins can be used to create 3D-printed implants with embedded microfluidic channels, enabling the rapid detection of specific biomarkers associated with various diseases, such as cancer or infectious diseases. By incorporating 4D-printed materials that respond to the presence of target molecules, these implants can provide highly sensitive and specific detection, enabling early diagnosis and timely intervention. For example, a 4D-printed implant with integrated microfluidic channels could change its shape or colour upon binding to a specific cancer biomarker, allowing for rapid and easy visualisation of the diagnostic result [83].

Additionally, 4D printing has the potential to revolutionise the field of medical imaging by creating anthropomorphic phantoms, which are physical models used to calibrate and validate imaging equipment for radiotherapy. By using 4D-printed materials that mimic the properties of human tissues and organs, researchers can create phantoms that accurately represent the patient’s unique anatomy, leading to more precise and personalised diagnostic imaging. For example, a 4D-printed deformable lung and liver phantom can be used to assess the accuracy of CT and MR imaging in radiotherapy planning. This enables clinicians to optimise treatment plans by accounting for organ motion during respiration, ultimately resulting in improved patient outcomes and reduced radiation exposure [84].

#### 2.2.6. Rehabilitation Devices

The advent of 4D printing technology has brought about a paradigm shift in the field of biomedical rehabilitation, introducing a new dimension of possibilities for creating adaptive, responsive, and smart devices. This new approach has the potential to redefine the design, manufacturing, and application of biomedical rehabilitation devices, making them more personalised, efficient, and comfortable for patients.

One example of 4D printing in rehabilitation is the creation of dynamic splints and braces for the management of musculoskeletal conditions. These smart devices can be programmed to change their shape or stiffness in response to muscle activation or joint position, offering customised support and promoting healing while minimising the risk of joint stiffness or muscle atrophy. For instance, researchers showed that a 4D-printed wearable system inspired by biological motion mechanisms can automatically adapt to provide dynamic support during specific tasks such as gripping or typing [85]. In another study, a semi-rigid wearable device that incorporates shape-memory and self-fusing properties has been developed, which allows the creation of customisable support systems for patients with musculoskeletal conditions [86].

4D printing can also be used for developing devices that emulate the characteristics of biological tissues, allowing for seamless integration with the human body and providing a natural, comfortable interface for rehabilitation purposes. One notable example is a 4D-printed prosthetic breast for patients who have undergone mastectomy surgery. By mimicking the natural properties of human tissues, this innovative prosthetic would adapt to the wearer’s body shape and movement, providing comfort and support during the recovery process. Moreover, the 4D-printed prosthetic could be designed to respond to changes in the wearer’s body temperature or muscle contractions, ensuring a dynamic fit and personalised experience. This approach not only enhances the wearer’s mobility and confidence, but also reduces the risk of complications associated with traditional prosthetics [87].

## 3. Challenges in 4D Printing for Biomedical Engineering

Despite the significant advancements in 4D printing for biomedical applications, several challenges remain to be addressed before the widespread adoption and implementation of this technology in clinical settings.

### 3.1. Materials Limitations

#### 3.1.1. Material Properties

The performance of 4D-printed biomedical devices and structures is heavily dependent on the materials used. Smart materials, such as shape-memory polymers, hydrogels, and stimuli-responsive materials, need to exhibit the desired behaviour and response to external stimuli to fulfill their intended function. However, finding materials with the appropriate properties can be challenging. The mechanical properties, such as strength, flexibility, and durability, of these materials may not always meet the requirements for specific biomedical applications. Additionally, it is often difficult to combine multiple smart materials in a single 4D-printed structure, limiting the range of functionalities that can be achieved.

Furthermore, the 4D printing process can also impose constraints on the materials used. For instance, some fabrication techniques require materials to withstand high temperatures, mechanical stress, or exposure to light or chemicals during the printing process. These factors can limit the range of materials available for 4D printing in biomedical applications.

#### 3.1.2. Biocompatibility

Biocompatibility is a critical consideration in biomedical engineering, as the materials used must be non-toxic and non-immunogenic to ensure the safety of patients [88,89]. This requirement can further limit the choice of materials for 4D printing. The development of biocompatible smart materials is still an ongoing area of research, and it can be challenging to find materials that not only exhibit the desired shape-changing behaviour, but also comply with the stringent biocompatibility requirements.

Moreover, the processing techniques used in 4D printing can also affect the biocompatibility of the final product. For example, some fabrication methods may leave residual chemicals or particles that could cause adverse reactions in patients. Ensuring that the 4D printing process does not compromise the biocompatibility of the printed structure is crucial for successful implementation in biomedical applications.

#### 3.1.3. Degradation Rate

The degradation rate of 4D-printed biomedical devices is a significant concern, particularly in applications such as drug delivery systems, tissue engineering, and implantable devices. The materials used in these applications should degrade at an appropriate rate, allowing the device or structure to perform its intended function without causing harm to the patient. For example, in drug delivery systems, the degradation rate should be controlled to ensure the proper release of the therapeutic agent over a specific period [15]. In tissue engineering, the scaffold material should degrade at a rate that matches the growth and development of new tissue [90].

However, achieving the desired degradation rate can be challenging due to the complex interactions between the material, the fabrication process, and the biological environment. Factors such as material composition, structure, and processing conditions can significantly affect the degradation behaviour of 4D-printed devices. Furthermore, the degradation rate may also be influenced by factors specific to the patient, such as the local biological environment, immune response, and individual healing rate. Researchers need to consider these variables when designing 4D-printed biomedical devices to ensure optimal performance and patient safety.

To overcome materials limitations in 4D printing for biomedical applications, several potential solutions and strategies can be employed. One approach is the development of novel biomaterials specifically designed for 4D printing, taking into account the unique requirements of the printing process and the desired functionality of the final biomedical device. Researchers can explore the synthesis and characterisation of biocompatible materials with enhanced mechanical properties, shape-memory behaviour, and responsiveness to external stimuli. Another strategy is the utilisation of composite materials, combining different components such as polymers, metals, ceramics, or bioactive agents to achieve desired properties and functionalities. Composite materials can enhance the mechanical strength, biocompatibility, and bioactivity of printed structures. Additionally, optimising the printing process parameters and techniques, such as print speed, temperature, and layer thickness, can improve the accuracy, resolution, and reproducibility of the printed biomedical devices. Thorough investigation and understanding of the interactions between the materials, printing process, and the intended application are crucial to overcome materials limitations and ensure the successful implementation of 4D printing in biomedical engineering. Continuous research and collaboration between materials scientists, engineers, and biomedical professionals are essential in driving advancements and addressing the challenges associated with materials limitations in 4D printing for biomedical applications.

### 3.2. Fabrication Complexities

#### 3.2.1. Integration of Multiple Materials

One of the key advantages of 4D printing is the ability to create structures with varying mechanical properties and responsiveness to stimuli by incorporating different materials into the printed object. This capability is particularly important in biomedical applications, where devices often need to exhibit a range of properties, such as flexibility, rigidity, or responsiveness to specific stimuli, to function effectively. However, integrating multiple materials in a single 4D-printed structure can be challenging due to the limitations of current printing technologies.

Achieving precise control over the distribution and interaction of different materials in a 4D-printed structure is essential to ensure the desired performance and behaviour of the final product. This requires advanced printing techniques that can deposit multiple materials simultaneously while maintaining precise control over their placement and interaction. Additionally, the materials used must be compatible with one another to prevent potential issues, such as delamination or poor adhesion between layers. Addressing these challenges will require the development of new printing techniques and material formulations specifically designed for multi-material 4D printing in biomedical applications.

#### 3.2.2. Resolution and Accuracy

High resolution and accuracy are crucial in 4D printing for biomedical engineering, as the printed structures often need to have intricate features or precise dimensions to function effectively. For example, tissue engineering scaffolds require a high degree of porosity and precise pore geometry to promote cell attachment and growth [91], while implantable devices must have accurate dimensions to fit properly within the patient’s body [39]. However, achieving the required resolution and accuracy in 4D printing can be challenging due to the limitations of current printing technologies.

Many 4D printing techniques struggle to achieve the resolution and accuracy needed for some biomedical applications, particularly when printing with smart materials that can change their shape or properties in response to external stimuli. Factors such as material viscosity, print speed, and printer hardware can affect the resolution and accuracy of the printed structure. Researchers need to develop new printing technologies and optimise printing parameters to achieve the required resolution and accuracy for specific biomedical applications.

#### 3.2.3. Scalability

Scalability is another significant challenge in 4D printing for biomedical engineering, as the printed structures must often be produced in large quantities or at different scales to meet the needs of various patients. Moreover, the manufacturing process should be cost-effective and time-efficient to ensure the widespread adoption of 4D-printed biomedical devices. However, many current 4D printing techniques are time-consuming and have limited scalability, making them unsuitable for large-scale production.

Addressing the issue of scalability in 4D printing will require the development of new printing techniques that can produce structures at a faster rate and with minimal material waste. Additionally, researchers need to explore methods for automating the 4D printing process and integrating it with other manufacturing technologies, such as injection moulding or additive manufacturing, to increase production capacity and reduce costs.

### 3.3. Regulatory and Ethical Considerations

#### 3.3.1. Regulatory Compliance

The introduction of any new medical technology, including 4D printing, requires rigorous testing and approval from regulatory agencies to ensure the safety and efficacy of the devices and structures being produced. In many countries, agencies such as the US Food and Drug Administration (FDA) or the European Medicines Agency (EMA) oversee the regulation of medical products, including those manufactured using 4D printing techniques.

Navigating the complex regulatory landscape can be challenging, as the approval process for 4D-printed biomedical devices often involves multiple stages, including preclinical testing, clinical trials, and post-market surveillance. Moreover, given the novelty of 4D printing technology, there may be gaps in existing regulations or the need for new guidelines specifically tailored to address the unique aspects of 4D-printed devices, such as their adaptability and shape-changing properties.

Researchers and manufacturers need to work closely with regulatory agencies to develop a clear understanding of the requirements and approval processes for 4D-printed biomedical devices. This includes ensuring that the materials used are biocompatible, the fabrication processes meet quality standards, and the devices demonstrate safety and efficacy in preclinical and clinical testing.

#### 3.3.2. Ethical Compliance

The implementation of 4D printing in biomedical engineering raises several ethical questions that must be carefully considered to ensure the responsible development and use of this technology. Some key ethical considerations include patient privacy, informed consent, and equitable access to 4D-printed devices.

Patient privacy is a crucial concern, as the development of customised 4D-printed devices often requires access to personal medical data, such as patient-specific anatomy or physiological parameters. Researchers and manufacturers need to develop strategies for protecting patient privacy and ensuring the secure handling of sensitive data throughout the design and manufacturing process.

Informed consent is another important ethical consideration, particularly in the context of clinical trials involving 4D-printed devices. Patients participating in such trials should be fully informed of the potential risks and benefits associated with the use of 4D-printed devices, as well as any alternatives to their use.

Equitable access to 4D-printed biomedical devices is also a concern, as the high costs associated with the development and manufacturing of these devices may limit their availability to patients in need. Researchers and policymakers need to explore strategies for reducing the costs of 4D printing technologies and ensuring that the benefits of this innovative approach to healthcare are accessible to all, regardless of their socioeconomic status.

In addition to the general limitations of 4D printing in biomedical engineering mentioned above, it is important to recognise that each specific application within this field may entail its own unique set of challenges. The requirements and objectives of different projects vary greatly, necessitating tailored approaches and problem-solving strategies. Each combination of materials, techniques, and applications presents its own set of challenges that necessitate thorough investigation. Factors such as the desired functionality, compatibility with biological systems, structural complexity, and scalability can all introduce specific hurdles that need to be thoroughly investigated. Therefore, it is essential to approach each application individually, conducting comprehensive research and analysis to identify and address the specific challenges associated with that particular project. By doing so, we can effectively navigate the limitations of 4D printing and unlock its full potential in the diverse landscape of biomedical engineering.

## 4. Future Directions for 4D Printing in Biomedical Engineering

As 4D printing technology continues to mature, there is immense potential for further advancements in biomedical engineering. This section highlights future directions for 4D printing in the field, offering novel solutions to pressing clinical challenges.

### 4.1. Emerging Trends and Areas of Research

4D printing involves the creation of smart materials that can change their properties or shape over time in response to external stimuli. As research and development in this field progress, several emerging trends and areas of research are expected to shape the future of 4D printing in biomedical engineering.

#### 4.1.1. Integration of Sensors and Electronics

One promising area of research in 4D printing involves the integration of sensors and electronics into the printed structures [19]. By embedding sensors and electronic components within 4D-printed devices, it becomes possible to monitor the performance of the devices in real-time, gather valuable data on patient health, and enable responsive behaviour. For instance, 4D-printed implants with integrated sensors could monitor the healing process, detect potential complications, and provide feedback to healthcare providers, enabling more personalised and effective treatment plans.

#### 4.1.2. Biohybrid Systems

Combining living cells with 4D-printed structures can lead to the development of biohybrid systems that exhibit the desired characteristics of both biological tissues and engineered materials. Researchers are exploring the possibility of creating 4D-printed constructs with living cells [92], which can grow and adapt in response to their environment, paving the way for novel applications in tissue engineering and regenerative medicine. This approach could potentially reduce the need for organ transplantation by providing functional alternatives made from a patient’s own cells.

#### 4.1.3. Self-Healing Materials

The development of self-healing materials for 4D printing is another exciting area of research that could have significant implications for biomedical engineering. By incorporating self-healing properties into 4D-printed devices, researchers can create structures that can repair themselves in response to damage, extending their lifespan and improving their reliability [6]. This could be particularly beneficial in applications such as implants or prosthetics, where the ability to self-repair would reduce the need for replacement or invasive surgeries.

#### 4.1.4. Personalised Medicine

4D printing has the potential to play a significant role in the growing field of personalised medicine. By enabling the fabrication of patient-specific devices and structures, 4D printing can help tailor treatments and interventions to the unique needs of individual patients. This includes the development of customised implants and prosthetics that fit the patient’s anatomy perfectly [77], as well as patient-specific drug delivery systems that ensure optimal dosing and targeted delivery of therapeutic agents [93]. The widespread adoption of 4D printing in personalised medicine could lead to more effective treatments and improved patient outcomes.

#### 4.1.5. Environmental and Biodegradable Materials

As concerns over the environmental impact of materials and waste disposal grow, there is increasing interest in developing environmentally friendly and biodegradable materials for 4D printing in biomedical engineering. Researchers are exploring the use of naturally derived materials, such as cellulose or chitosan, as well as biodegradable synthetic polymers for 4D printing applications [94]. The use of these materials could help reduce the environmental impact of biomedical devices and contribute to more sustainable healthcare practices.

#### 4.1.6. Multi-Stimuli Responsive Materials

Researchers are investigating the potential of multi-stimuli responsive materials in 4D printing for biomedical applications. These materials can respond to multiple external stimuli, such as temperature, light, pH, or magnetic fields, and exhibit different behaviours depending on the specific stimulus. This adaptability can lead to the development of 4D-printed devices and structures with enhanced functionality and versatility. For instance, a drug delivery system made from multi-stimuli responsive materials could adjust the release rate and targeting of therapeutic agents based on the patient’s body temperature, pH, or other biological factors, resulting in more effective and personalised treatments [95].

#### 4.1.7. Biomimetic Materials

The field of biomimicry, which involves the imitation of natural systems and structures, has inspired the development of biomimetic materials for 4D printing in biomedical engineering. These materials mimic the properties and functions of biological tissues, providing potential advantages in terms of biocompatibility, functionality, and adaptability. Researchers are exploring the use of biomimetic materials in various applications, including tissue engineering, regenerative medicine, and the fabrication of implants and prosthetics that closely resemble the patient’s native tissues. By incorporating biomimetic materials into 4D-printed structures, researchers can create devices that integrate seamlessly with the body and promote more effective healing and recovery [96,97].

#### 4.1.8. Nanomedicine

The focus in this area will be on the design, synthesis, and application of materials and devices at the nanoscale. Advancements in 4D printing techniques will enable the creation of sophisticated, responsive nanostructures and drug-delivery systems that target specific cells, tissues, or physiological conditions. These nanoscale constructs can improve therapeutic efficacy, reduce side effects, and enable personalised medicine [98]. As research progresses, the integration of 4D printing technology with nanomedicine will offer promising new solutions for disease diagnosis, treatment, and prevention, revolutionising healthcare and enhancing patient outcomes.

### 4.2. Advancements in Fabrication Techniques

Advancements in fabrication techniques are crucial for driving innovation in 4D printing and other emerging technologies. As the field evolves, researchers continue to explore new methods and refine existing ones to create more sophisticated, efficient, and precise fabrication processes. Some notable advancements in fabrication techniques include:

#### 4.2.1. Multi-Material and Multi-Process Printing

Multi-material and multi-process printing is a pivotal advancement in fabrication techniques, enabling the creation of complex, multifunctional structures by combining different materials and processes simultaneously. This innovation is particularly significant for 4D printing, where materials with diverse responsiveness to stimuli are often merged to achieve desired dynamic behaviours. Multi-material printing allows the design of objects with distinct regions exhibiting varying mechanical, thermal, or electrical properties, broadening the scope for advanced materials that respond to specific environmental stimuli. Multi-process printing enhances this concept by integrating multiple fabrication techniques within a single build, generating structures with diverse fabrication-induced characteristics. This progress in multi-material and multi-process printing has impacted 4D printing applications in biomedical engineering, soft robotics, and smart materials. Overcoming remaining challenges, such as advanced printing systems and material compatibility, will further expand the potential of 4D printing technology [99,100].

#### 4.2.2. High-Resolution Printing

High-resolution printing has made significant strides in fabrication techniques, allowing for the creation of structures with intricate geometries and features at the micro- or even nanoscale. This precision is crucial for applications such as tissue engineering and drug delivery systems, where fine control over structure and material properties is essential. Developments in high-resolution printing techniques, such as two-photon polymerisation [101], have expanded the possibilities for fabricating complex 3D- and 4D-printed constructs, enabling a higher level of detail and functionality. This has led to the creation of more sophisticated medical devices, biomimetic tissues, and targeted drug delivery systems, among other applications. As research continues, further advancements in high-resolution printing techniques will drive innovation in various fields, including biomedical engineering and nanotechnology, enhancing the potential impact and capabilities of 4D printing and other emerging technologies.

#### 4.2.3. Hybrid Fabrication Techniques

Hybrid fabrication techniques, which combine multiple fabrication methods, have emerged as a key advancement in fabrication technology. These techniques unlock novel structures and functionalities by merging methods such as 3D printing with electrospinning or bioprinting with microfluidics. This synergy results in 4D-printed constructs with enhanced mechanical, biological, and stimuli-responsive properties. These hybrid techniques can create constructs with diverse characteristics, paving the way for new applications in biomedical engineering. For example, combining 3D printing with electrospinning can produce scaffolds with tunable mechanical properties and tailored pore structures, which are ideal for tissue engineering [102,103]. Continued research into hybrid fabrication techniques will further expand the capabilities and potential applications of 4D printing, leading to novel innovations in various fields.

#### 4.2.4. Self-Assembly and Self-Folding Techniques

Self-assembly and self-folding techniques are emerging as promising fabrication methods for creating complex 3D and 4D structures. By leveraging the inherent properties of materials and precise control of environmental conditions, these techniques enable the formation of dynamic constructs without the need for external manipulation or assembly. Self-assembly relies on the spontaneous organisation of materials into ordered structures, driven by molecular interactions and surface chemistry. Self-folding, on the other hand, involves inducing shape changes in materials through programmed stress patterns or stimuli-responsive elements [15,104,105]. Both approaches are vital for 4D printing applications, as they can produce structures that adapt and respond to environmental cues. Advancements in self-assembly and self-folding techniques will broaden the possibilities for 4D printing in biomedical engineering, contributing to the development of innovative solutions and technologies.

#### 4.2.5. Embedded Sensors and Actuators

Embedded sensors and actuators in 4D-printed constructs offer real-time monitoring and control of their behaviours, enabling the creation of smart structures that respond to various stimuli [19,106]. Advances in fabrication techniques allow for the seamless integration of these components into printed constructs, enhancing their functionality. For instance, embedded sensors in tissue-engineered constructs can provide continuous monitoring of cellular activities. Further research in embedding sensors and actuators will unlock new possibilities in 4D printing, leading to innovative solutions across various domains of biomedical engineering.

#### 4.2.6. Biofabrication

Biofabrication techniques, such as bioprinting [70] and cell-laden hydrogel printing [66], have advanced significantly, enabling the incorporation of living cells and biomolecules within 3D- and 4D-printed constructs. This is essential for applications like tissue engineering and regenerative medicine, where interactions between printed materials and living cells are critical for success. Advancements in biofabrication have led to the creation of more biomimetic and functional tissue constructs, improving their integration within the body and promoting healing [107]. As research progresses, biofabrication techniques will continue to evolve, allowing for more sophisticated 4D-printed constructs with enhanced biological properties, ultimately revolutionising healthcare and providing new therapeutic options.

#### 4.2.7. Machine Learning and AI-Driven Design

Machine learning and AI-driven design have become increasingly important in the development and optimisation of fabrication processes, including 4D printing. These technologies can predict the behaviour of 4D-printed materials and structures, guiding the selection of materials, geometries, and fabrication parameters to achieve desired functionalities. Incorporating AI and machine learning into the design process allows for the exploration of vast design spaces, identification of optimal solutions, and prediction of material properties and performance. This leads to more efficient and effective fabrication processes and improved 4D-printed constructs. As research in AI and machine learning progresses, their integration into fabrication techniques will enhance the capabilities of 4D printing technology, enabling the creation of increasingly complex, functional, and responsive materials and structures across various applications and industries [108,109,110].

#### 4.2.8. Stability and Automation

Scalability and automation are essential for translating 4D printing from research labs to real-world applications. Advancements in high-throughput manufacturing methods, automated quality control systems, and standardised processes and protocols will accelerate the large-scale production of 4D-printed constructs. By improving scalability and automation, 4D printing technology can become more accessible and cost-effective, paving the way for widespread adoption in biomedical engineering. As research continues, further innovations in scalable and automated fabrication techniques will play a crucial role in unlocking the full potential of 4D printing and driving its integration into various applications.

## 5. Conclusions

This review paper has provided a comprehensive overview of the advancements, challenges, and future directions for 4D printing in biomedical engineering. 4D printing technology has emerged as a promising approach to address various unmet clinical needs and revolutionise healthcare practices by integrating the advantages of 3D printing with the added dimension of time-dependent shape transformations.

The paper has discussed the significant advancements in 4D printing for biomedical applications, including the development of stimuli-responsive materials, innovations in fabrication techniques, and successful implementation in various fields such as tissue engineering, drug delivery, and medical devices. Moreover, the review has highlighted several exciting future directions for 4D printing in biomedical engineering, such as the development of advanced smart materials, integration of bioprinting and 4D printing techniques, personalised medicine, nanomedicine, and bioelectronic devices. Addressing these challenges and exploring these future directions will be essential for realising the full potential of 4D printing technology in various biomedical applications.

As 4D printing technology continues to advance, it is poised to make significant contributions to biomedical engineering, ultimately improving patient outcomes and transforming healthcare practices. By fostering interdisciplinary collaboration, promoting public engagement and education, and encouraging industry partnerships, 4D printing has the potential to transform the way we approach the design and development of medical devices, treatments, and therapies. It is clear that 4D printing technology holds immense promise for the future of biomedical engineering, and continued research and innovation in this field will pave the way for innovative advancements in healthcare.

## Figures and Tables

**Table 1:** Comparison of 3D and 4D Printing Technologies.

Feature	3D Printing	4D Printing
Principle	Layer-by-layer fabrication of static structures	Layer-by-layer fabrication with embedded shape-changing properties
Material Options	Plastics, metals, ceramics, composites	Shape-memory polymers, hydrogels, stimuli-responsive composites, metals, ceramics
Complexity	Limited to static shapes and structures	Dynamic structures with time-dependent shape transformations
Biomedical Applications	Prosthetics, implants, tissue scaffolds, medical devices	Smart drug delivery systems, tissue engineering, soft robotics, self-deploying implants, etc.
Advantages	Customisation, geometric complexity, reduced waste	Added functionality, adaptability, responsive behaviour
Limitations	Restricted to static structures, limited stimuli-responsive materials	Complex design process, limited material options, potential biocompatibility concerns
